# Parenting with psychosis: An exploration of the everyday parenting practices of parents with psychotic disorders

**DOI:** 10.1111/papt.70031

**Published:** 2025-12-22

**Authors:** Hannah Collins, Anja Wittkowski, Lauren Wolfenden, Rachel Calam, Lynsey Gregg

**Affiliations:** ^1^ Division of Psychology and Mental Health, School of Health Sciences University of Manchester Manchester UK; ^2^ Greater Manchester Mental Health NHS Foundation Trust Manchester UK; ^3^ Manchester Academic Health Science Centre Manchester UK

**Keywords:** parenting style, qualitative, schizophrenia, serious mental illness

## Abstract

**Objectives:**

Parental psychosis poses challenges for families but tailored support for parents remains limited. Very few studies have explored the day‐to‐day parenting practices of parents with psychosis in depth, and none have qualitatively compared their parenting to that of parents without serious mental illness (SMI). This study compared accounts from parents with psychosis to accounts from parents without SMI to explore parenting practices and identify areas for targeted support.

**Design and Methods:**

Semi‐structured qualitative interviews were used to explore the daily reality of child‐rearing in 20 parents with psychosis and 20 parents without serious mental illness. Data were analysed thematically using Template Analysis.

**Results:**

Three a priori themes were deductively generated from an established parenting model: *Positive authoritative parenting*, *Negative authoritarian parenting* and *Permissive/avoidant parenting*. Both groups of parents used positive parenting strategies well, but challenges such as stress and fatigue made it difficult for parents in both groups to maintain this approach consistently. Parents with psychosis were more likely to utilise authoritarian and permissive and avoidant parenting styles due to feeling overwhelmed, subsequently accompanied by feelings of guilt which may in turn, impact their mental health.

**Conclusions:**

The present study adds novel insights into how additional challenges such as positive symptoms and fatigue can affect the parenting behaviours of parents with psychosis resulting in greater use of maladaptive parenting practices. Evidence‐based parenting courses and utilisation of family‐focused approaches within services are recommended for families affected by parental psychosis to improve outcomes for parents and their children.

## INTRODUCTION

Around 440,000 adults within the UK experience psychosis (McManus et al., 2016) and over a third are also parents (Radley, Sivarajah, et al., 2022). Psychosis can involve a loss of contact with reality, hallucinations, delusions or altered thought processes (American Psychiatric Association, 2013), experiences that can impact a parent's ability to cope with the demands of child‐rearing or decrease their satisfaction with the parental role (Campbell et al., 2018; Strand et al., 2020). Although many parents with psychosis cope well with parenting (Campbell et al., 2012; 2018) and being a parent is an important step towards recovery (van der Ende et al., 2016), some parents experience significant difficulties and find that the stress of parenting can lead to an exacerbation of psychotic symptoms (Evenson et al., 2008; Radley et al., 2023).

Research has shown that parents with psychosis experience difficulties in establishing family routines, maintaining boundaries and asserting discipline (Strand et al., 2020). They have been reported to demonstrate more hostility and criticism towards their children and make more child‐blaming attributions than parents without SMI (Gregg et al., 2021). Providing protection, support and comfort for their child(ren) has been reported to be particularly difficult when positive psychotic symptoms such as hallucinations and delusions are present (Healy et al., 2016; Strand et al., 2020). A recent systematic review of 12 studies by Collins et al. (2025) highlighted that stress and fatigue also affect parenting. Parents with psychosis are more likely to use permissive and avoidant parenting styles when feeling stressed or fatigued.

Despite the difficulties parents with psychosis experience, and the known impact of parental mental illness on the long‐term outcomes of children (e.g. Argent et al., 2020), very few studies have explored how parents with psychosis parent in day‐to‐day life. The review by Collins et al. (2025) found only three studies that had explored parenting in any depth qualitatively, and none had utilised a non‐clinical comparison group to explore similarities and differences in parenting. Thus, our understanding of parenting practices and support needs among parents with psychosis remains insufficient (Bee et al., 2014), limiting advancements in evidence‐based practices (Skivington et al., 2021).

This lack of research is reflected in the limited tailored support available for parents with psychosis (David et al., 2011; Day et al., 2011; Engur, 2017). Systemic approaches such as family‐focused practice, known to mitigate the risks for parents with SMI and their children (Foster et al., 2016; Grant et al., 2019; Maybery et al., 2015), have not been adequately implemented (Furlong et al., 2021) due to the difficulties associated with integrating such interventions into service provision (Tuck et al., 2023). A recent Cochrane review (Radley, Grant, et al., 2021) reported that a limited number of parenting interventions are available, but none have an evidence base for parents with psychosis. Since then, a case series has highlighted the potential benefits of a positive parenting program (called Triple P) for parents with psychosis (Wolfenden et al., 2022), but it has not been formally evaluated.

To improve parenting outcomes and support for parents with psychosis, an exploration of daily parenting practices, highlighting both strengths and difficulties, is imperative. In order to understand whether parents with psychosis are employing the same parenting strategies as parents without psychosis, a study involving both sets of parents is required. This is the first study to provide an in‐depth exploration of parenting in both groups.

## METHOD

### Design

This qualitative study, which utilised both secondary and primary data, used Template Analysis (Brooks et al., 2015) to explore parenting practices in parents first interviewed by Gregg et al. (2021) using the Camberwell Family Interview (CFI), supplemented with additional interview data to achieve a matched non‐clinical sample.

### Ethical approval

Originally, ethical approval was obtained from Greater Manchester West National Ethics Committee (15/NW/0532) and subsequently from the University of Manchester Research Ethics Committee (2022‐15067‐25321).

As the two samples in the original study were not well matched, with significant disparities observed between the two groups in terms of employment, marital status and financial difficulty, additional parents without SMI were recruited and interviewed to replace nine participants from the original data set and improve the match. Thirty‐one participants (20 parents with psychosis and 11 parents without SMI) from the original dataset were retained and utilised within the current study.

### Participant recruitment

In the original study by Gregg et al. (2021), parents with psychosis were recruited from Community Mental Health Teams and Early Intervention Services from four NHS Trusts across Greater Manchester, UK. They were required to meet ICD‐10 criteria for schizophrenia, schizotypal and delusional disorders (F10–F29), corroborated with a checklist and case note review. To protect participants from harm and reduce distress, participants were excluded if they had recently been in‐patients or if they were at risk of their child(ren) being removed from their care.

The original study by Gregg et al. (2021) recruited parents without SMI through a research volunteer database at the University of Manchester and within the voluntary sector and social services (including Local Authority Family Services). Additional parents recruited to improve the matching of non‐clinical parents to the parents with psychosis were recruited via local primary schools in the same geographical area as the original study, using school newsletters and posters.

Participants in both groups needed to be 18 years old or over and a parent or primary carer to a child aged 3–11 years, with whom they lived. Parents with multiple eligible children were asked to nominate an index child they reported experiencing the greatest challenges with. For participants to complete the interviews and provide consent, proficiency in English was also a requirement.

### Measures and procedure

All participants completed the *Family Background Questionnaire* (Sanders & Morawska, 2010a, 2010b), which collected demographic information, including parent and child age, gender, ethnicity and parental marital and employment status, education level and financial stress.

Participants were interviewed using a modified version of the *Camberwell Family Interview* (CFI, Vaughn & Leff, 1976, adapted by Peters et al., 2005). The CFI is a standardised, semi‐structured interview designed to assess the expressed emotion of relatives of family members with schizophrenia. Peters et al. (2005) modified the CFI to be relevant for the parents of young children (3–10 years old). This version of the CFI explores key areas of family life via a ‘family time budget’ focused on daily routines (e.g., mealtimes, schooling, and bedtime), irritability and conflict in the household; the child's behaviour; how the parent manages the child's behaviour and the impact of the child's behaviour on the parent. As such, it gives a good indication of the daily reality of caring for a child and the situations that the parent finds most challenging. Child behaviours specifically asked about in the CFI include aggression, destructive behaviour, tantrums, lies, fears and worries, under and overactivity. Indirect prompts are used to explore and reveal other possible areas of parental concern, for example, ‘*do you ever nag or grumble at [child]?*’. Training in the administration of the CFI was provided by a member of the research team who had been formally trained by one of the original authors of the CFI. A different team member contributed to the adaptation of the CFI (Peters et al., 2005) and confirmed its suitability for use with children aged 11.

Interviews took place in the participant's home (*n* = 31) in 2017 or online via Zoom or Microsoft Teams (*n* = 9) in 2022–2023. Informed consent was obtained. Interviews were conducted by authors 1 and 3 (LW and HC). Interviews were audio‐recorded, transcribed verbatim for analysis and pseudonymised. All parents were given a £20 voucher as a thank you for their participation. When interviews raised concerns about the parent or child's well‐being, these were reported to the appropriate agencies (typically keyworkers).

### Data analysis

The first author (HC) read through all 40 transcripts several times to ensure content familiarisation. Transcripts were imported into NVivo 14 (Lumivero, 2023) before conducting thematic analysis using Template Analysis (Brooks et al., 2015) utilising both deductive and inductive approaches. Three top‐level a priori themes were deductively generated from Baumrind's (1966) model of parenting and associated measures of parenting, for example, The Parenting Styles and Dimensions Questionnaire, Robinson et al. (2001) and the Parenting and Family Adjustment Scales, Sanders and Morawska (2010a, 2010b). Inductive coding was conducted to expand upon the a priori themes based on the raw data (Thomas, 2006).

An initial template was created using the first set of 10 interviews, with five transcripts from each parent group. Next, a further five interviews were mapped onto the initial template, adjusting the template as needed to ensure all relevant data were appropriately coded. To conclude the process, the final version of the template was applied to the remaining interview data, and themes were redefined or removed if they did not prove to be useful or meaningful for the dataset. The final template is presented in Table 1.

**TABLE 1:** Final template of top‐level themes and sub‐themes.

Positive Authoritative parenting	
1.1 Connection and warmth	1.1.1 Offering of affection and warmth
	1.1.2 Engagement in meaningful conversations
	1.1.3 Responsive to child's needs and feelings
	1.1.4 Spending time with child
1.2 Regulation and reasoning	1.2.1 Clear rules and boundaries
	1.2.2 Prompts/reminders and encouragement
	1.2.3 Explanation of rules/boundaries and rationale
	1.2.4 Discussions on impact of behaviour
1.3 Autonomy	1.3.1 Respect and encouragement of child's opinions and choices
	1.3.2 Allows child to give input into family rules
	1.3.3 Child's desires taken into account
1.4 Positive encouragement	1.4.1 Treat, reward, attention or fun activity for behaving well
	1.4.2 Praises child
1.5 Parental consistency	1.5.1 Predictable and stable approach
	1.5.2 Structure and routine
2 Negative Authoritarian Parenting	
2.1 Physical coercion	2.1.1 Physical punishment
	2.1.2 Restraint
2.2 Verbal hostility	2.2.1 Yells, shouts or swears at child
	2.2.2 Critical of child/ name‐calling
2.3 Non‐reasoning/punitive	2.3.1 Isolates child
	2.3.2 Uses threats or punishes child without explanation
	2.3.3 Ignores child
3 Permissive/Avoidant Parenting	
3.1 Indulgence	3.1.1 Little or no rules or boundaries
	3.1.2 Ignores behaviour
	3.1.3 Gives in to child
3.2 Withdrawal	3.2.1 Avoidance/withdrawal from disciplining child
	3.2.2 Takes self away from situation
	3.2.3 Difficulty coping

T‐tests and chi‐squared tests were carried out to test for group differences in key demographic variables to determine if the two groups were adequately matched.

Three authors (LW, LG and RC) were involved in the primary study that this study is secondary to, but the researcher who coded the data and determined the themes for the present study (HC) was not. Another researcher not involved in the initial study was added to the research team to bring a fresh perspective (AW). These steps were taken to reduce the limitations inherent in secondary data analysis of existing qualitative data (Ruggiano & Perry, 2019).

### Reflexivity statement

Recognising that qualitative research is shaped by the subjectivity of the researcher (Smith et al., 2009), reflexivity was embedded throughout the research process. All researchers were from white European backgrounds and held professional experience in academic and/or clinical psychology roles, with a particular focus on parenting and parental mental health. The initial template was informed by a broad review of parenting frameworks and refined as the lead researcher engaged more deeply with the data, alongside regular supervision. During supervision, a second researcher (LG) reviewed and discussed the coding of two interviews, which supported the development and clarification of the emerging template. A third researcher, who had not been involved in earlier stages of analysis, reviewed and critically reflected on the developing themes. These iterative changes were documented and justified, in line with the strengths of template analysis, which encourages transparency around how themes are developed from participant accounts (Brooks et al., 2015). This process helped ensure that interpretations remained grounded in the data, rather than influenced by researcher subjectivity. Maintaining successive versions of the template, engaging in collaborative discussions, and recording written reflections on analytic decisions all supported the rigour and reflexivity of the study.

## RESULTS

### Participant characteristics

Participants in the clinical group had a diagnosis of schizophrenia (*n* = 11) or paranoid schizophrenia (*n* = 9). Table 2 provides an overview of key demographic characteristics and family circumstances for parents with psychosis and those without. Unlike the Gregg et al. (2021) study, in which these two groups of parents significantly differed in parental age and household composition, these differences were no longer present for the current sample. However, differences remained in employment status and financial difficulty: Parents with psychosis were more likely to be unemployed (χ^2^(2) = 15.87, *p* < .001) and were more likely to be unable to meet essential expenses (χ^2^(1) = 15.82, *p* < .001). There were no other differences between the two groups.

**TABLE 2:** Participant characteristics.

	Parents with psychosis (*n* = 20)	Parents without SMI (*n* = 20)
Parent sex, *N* (%)		
Female	19 (95%)	19 (95%)
Male	1 (5%)	1 (5%)
Parent ethnicity, *N* (%)		
White	15 (75%)	17 (85%)
Black	2 (10%)	2 (10%)
Chinese	1 (5%)	0 (0%)
South Asian	1 (5%)	1 (5%)
Mixed	1 (5%)	0 (0%)
Parent age, mean (range)	33.9 (25–50)	36.6 (28–51)
Number of children, mean (range)	2 (1–5)	2 (1–6)
Child's sex, *N* (%)		
Female	6 (30%)	10 (50%)
Male	14 (70%)	10 (50%)
Child's ethnicity, *N* (%)		
White	12 (60%)	15 (75%)
Black	1 (%)	2 (10%)
South Asian	1 (5%)	0 (0%)
Mixed	6 (30%)	3 (15%)
Child illness or disability, *N* (%)		
Chronic illness (e.g. eczema, asthma)	6 (30%)	6 (30%)
Developmental delay (e.g. autism)	7 (35%)	3 (15%)
Child's age, mean (range)	8 (3–11)	7 (4–11)
Household composition, *N* (%)		
Single parent household	15 (75%)	12 (60%)
Dual parent household	5 (25%)	8 (40%)
Parental employment, *N* (%)		
Unemployed	19 (95%)	7 (35%)
Part time employment	1 (5%)	11 (55%)
Full time employment	0 (0%)	2 (10%)
Unable to meet expenses at times, *N* (%)	19 (95%)	7 (35%)

Abbreviation: SMI, serious mental illness.

### Template analysis

A frequency count of the number of references included under each code used in the template is provided in Table 3. It reveals that both sets of parents reported using all three overarching parenting strategies. Positive authoritative parenting was the most frequently reported parenting approach used by parents in both groups. Examples of negative authoritative parenting and permissive/avoidant parenting were more frequently reported by parents with psychosis.

**TABLE 3:** Total number of references recorded for each top‐level theme and subtheme.

Theme/subtheme	Number of references recorded	
	Parents with psychosis	Parents without SMI
**1. Positive authoritative parenting**	413	620
1.1 Connection and warmth	143	221
1.2 Regulation and reasoning	60	108
1.3 Autonomy	35	38
1.4 Positive encouragement	35	51
1.5 Parental consistency	21	54
**2. Negative authoritarian parenting**	133	44
2.1 Physical coercion	8	2
2.2 Verbal hostility	65	17
2.3 Non‐reasoning/punitive	60	23
**3. Permissive/avoidant parenting**	132	54
3.1 Indulgence	96	29
3.2 Withdrawal	36	23

Abbreviation: SMI, serious mental illness.

#### Theme 1: Positive authoritative parenting

This theme, consisting of five subthemes, depicts a parenting style characterised by high levels of responsiveness to the child, alongside firm boundary setting. Parents described warmth and connection, setting consistent rules and boundaries, being responsive to their child's needs and nurturing their child, as well as validating their child's feelings and valuing their opinions. Positive authoritative parenting was the most prominent theme throughout the interviews, with parents from both groups reporting using this parenting style most often.

##### Subtheme 1.1: Connection and warmth

Both groups of parents described connection and warmth in their parental style. They provided comfort, displayed affection and showed interest in their child's daily life and emotions. Parents in both groups described having a close and warm connection with their child, frequently offering comfort and affection during times of distress, as well as showing love through ‘acts of service’: ‘he sat up in the middle of the night and I said, “what's wrong” and he said “I'm scared” but I put my arm round him and just cuddled up’ (P31, parent with psychosis), ‘when she's on the residential, I wrote little notes and wrapped them up in her socks, for when she opened up her socks and stuff’ (P5A, parent without SMI).

All parent interviews showed parents engaging with their children, discussing school, recognising fears, worries and shifts in their child's emotional state and initiating supportive conversations. Some parents proactively sought to understand their child's emotional needs and employed various creative and adaptive strategies, such as ‘chat time’ (P52, parent with psychosis) or encouraged their child to write down their emotions when they struggled to verbalise their feelings (P22, parent without SMI). While both groups of parents responded to their child's needs and engaged their child in conversations, parents without SMI reported showing responsiveness to their child's emotional needs more frequently.

Some parents without SMI described labelling their child's emotion as well as teaching emotional regulation techniques: ‘Since a young age I've kind of done the things “right, you're getting frustrated, we need to take a deep breath”… and do those deep breathing with him… he will occasionally now do those by himself’ (P2A, parent without SMI).

Parents with psychosis discussed some of their struggles with their mental health, but they described striving to remain positive and masking their difficulties in order to protect their child: ‘I'm not gonna tell him I'm not okay… and he just said mum you look sad… and I'll reassure him that I am okay because I don't want him to worry’ (P50, parent with psychosis). Despite their mental health difficulties, parents with psychosis described strong bonds with their children and aimed to maintain positivity and hide their own struggles to shield their children from worry.

##### Subtheme 1.2: Regulation and reasoning

Parents from both groups explained and established clear rules and boundaries for their children, while helping their child to understand the implementation of consequences. Parents utilised prompts, warnings and timers to help remind their child of these rules. For example, many parents used reminders to ensure their children completed tasks, such as getting ready for school. Parents described using different disciplinary strategies, with the most common being ‘time out’, time in the child's bedroom or taking privileges away, such as removal of technology for a period: ‘when she does have her iPad… she just ignores what you're telling her to do… so I take her iPad off her’ (P53, parent with psychosis).

After applying consequences, parents typically engaged in conversations with their children, discussing feelings and the impact of their behaviour to enhance further understanding and reduce the likelihood of the child displaying the behaviour again in the future. Both groups acknowledged this to be a highly effective strategy, promoting a better understanding of rules and boundaries; however, a parent with psychosis also described the process as ‘draining’ and ‘exhausting’ (P50). Although used by both parents, this strategy was used more frequently in parents without SMI. Some parents saw this as a valuable learning opportunity, noting positive changes in their child's behaviour and understanding: ‘I really think the talking does help… she's quite a learner… if there's a lesson, she kind of tries to stick to it’ (P8A, parent without SMI). Another parent found it changed the meaning of the consequence for the child: ‘so now, when I do tell him something, sometimes he will do it because he knows that its right, it's not because he's listening to whatever I'm saying… he's agreeing with what I am saying’ (P50, parent with psychosis).

##### Subtheme 1.3: Autonomy

Both sets of parents promoted autonomy through encouraging their children to make choices, take responsibility and freely express their opinions and input into family life. Parents were more likely to have set structure and routines on weekdays but were more flexible and allowed more choice at weekends. Parents viewed this approach as empowering for the child because it increased their child's sense of control, motivation, and the likelihood of them completing a task: ‘I give him options… so I've worked really hard on trying to make him feel like he's in control… so, but talking to him and letting him feel like he's in control, like I've said, really helps’ (P9A, parent without SMI).

Both sets of parents recognised the importance of offering choices to their children because it not only empowered them but also facilitated discussions around family rules, allowing the child to have a sense of responsibility. For instance, one parent described compromising with her child on her bedtime: ‘we've reached a compromise of half eight… I want her to feel like she can have her say, so yeah, we've moved it to half eight, just so that she can feel like a small victory for her’ (P8A, parent without SMI).

All parents emphasised the significance of creating an environment in which their child felt comfortable expressing themselves and contributing their opinions, with some parents actively avoiding re‐creating their own childhood experiences of feeling ‘afraid’ to speak up (P35, parent with psychosis). Parents also expressed enthusiasm for understanding their child(ren)'s perspectives, valuing their insight and indicating a willingness to encourage open communication: ‘I don't mind that because I like getting to know what sort of person my child is at this present time, so I completely, am encouraging him to speak his mind’ (P50, parent with psychosis). Overall, this parenting approach aimed to allow their children to feel valued, heard and respected.

##### Subtheme 1.4: Positive encouragement

Parents across both groups described employing positive encouragement strategies, actively using praise, rewards and support to reinforce their child's positive behaviours and actions. Parents recognised the importance of verbal acknowledgment, praising their child for following instructions or behaving well: ‘I do praise him for it when he has been good and he's done as he was told, I say oh thank you well done you know’ (P32, parent with psychosis). Parents emphasised the significance of praising their child, believing that acknowledgement and appreciation would encourage further positive behaviour: ‘if S does something good, I said oh that's lovely you've done a good job there daughter, I said I'm really proud of you for doing that… so I praise it cos I think if I praise it she'll do it more’ (P19, parent without SMI).

Several parents across both groups implemented reward systems such as sticker charts and used incentives to motivate good behaviour: “if she's been good in the weekday… at the weekend I try and take her for a treat… you know a little treat just to say well done for being so good” (P42, parent with psychosis). Additionally, they prioritised praising honesty and truthfulness, valuing these aspects over potential mistakes or misbehaviour:

I'm so glad you told me, like you told the truth, so I'm not quite cross… she was so worried I was going to be cross… I was like, ‘no, I'm really proud of you for telling me (P5A, parent without SMI).

##### Subtheme 1.5: Parental consistency

Parental consistency involves parents maintaining a predictable and stable approach across different situations and over time. Although explicit mentions of consistent parenting were sparse in the interviews, both groups of parents stressed the implementation of a structured routine, particularly in the morning and before bedtime. However, routines were more frequently reported by parents without SMI.

Parents believed that a predictable routine contributed to their child's sense of security and positively influenced their behaviour: ‘I have noticed that since he's got the same routine every day, he does work well…’ (P1A, parent without SMI). Additionally, parents of children who struggled to regulate their emotions emphasised the importance of creating a stable and predictable environment for their child: ‘I think what he really needs is just stability and a routine, that's what he really needs… and I'm trying to give him that routine at home’ (P50, parent with psychosis).

#### Theme 2: Negative authoritarian parenting

This theme, with its three subthemes, is characterised by high expectations from parents and low responsiveness to a child's needs and emotions. Parents provided insights into their use of authoritarian methods, which included employing physical punishment, restraint, verbal aggression and use of punitive measures/punishments without further explanation or rationale. This theme was reported on much less frequently than positive authoritative parenting, indicating that parents from both groups are less likely to use this parenting style.

##### Subtheme 2.1: Physical coercion

In general, few parents employed physical coercion as a means of discipline or control, but a small subset in both groups reported occasionally resorting to it, particularly when parents were under time constraints or experiencing heightened stress. For example, one parent described an incident when an escalating conflict between their children led them to resort to physical intervention after exhausting other methods:

The last time it happened I ended up banging their heads together… and I just went like this and I just thought you know what BANG and all of a sudden it stopped (P49, parent with psychosis).

Parents described using different means of physical force, such as restraint, to calm their child or prevent behaviours from escalating when verbal communication proved ineffective: ‘I would say it's more of a restraining, because she can get in such a place where she just will not stop… oh I hate those days’ (P22, parent without SMI). When physical coercion was reported, parents instantly expressed feelings of guilt and acknowledged the inappropriateness of such practices.

I don't like doing that ‘cause of his reaction… he's like “how could you do that to me” and I was looking like thinking, ‘cause obviously you slap ‘em they cry then they look at you thinking “I hate you”… the first place my head went was “I hurt you” (P31, parent with psychosis).

Conversely, despite an initial instinct to use physical coercion another parent described how, due to changes in legislation regarding such practices, they have had to adopt new ways of disciplining their child:

Up until this new legislation came in if I lost my temper they knew to move because if they didn't, they'd get belted, literally I'd smack them and I wouldn't think twice about doing it, but obviously you're not allowed to do that anymore, so I've got to find ulterior ways of dealing with her (P49, parent with psychosis).

##### Subtheme 2.2: Verbal hostility

Verbal hostility, including instances of shouting and swearing at their child, was reported by both groups. Like physical coercion, parents acknowledged that they sometimes resorted to shouting when experiencing constraints like time pressure, emotional fatigue or when alternative methods had proved ineffective: ‘I try and keep my voice really calm… so I do that for as long as I can, and then ultimately I shout, I do shout’ (P5A, parent without SMI). Some parents described shouting as being the only method that worked: ‘you have to keep shouting at her, arguing with her, just to get her to do anything… and she will only listen to you if you are screaming your head off telling her to stop’ (P42, parent with psychosis).

Overall, parents from both groups expressed guilt and discomfort with this approach, considering it ineffective but sometimes struggling to control their reactions. One parent described sitting down with their child to explain their own behaviour: ‘I always make a point of after sitting with him and saying, it's not you, it's me, I'm very stressed out, I'm very tired, and I just reacted badly’ (P3A, parent without SMI). Both sets of parents were concerned about setting a poor example, leading to worries about their child adopting the same behaviour: ‘It's at a certain point he will shout back and that's when you kinda think okay I need to calm down now, because it's not setting a good example’ (P10, parent without SMI); ‘he hears me shouting at him, doesn't he so he's bound to shout’ (P35, parent with psychosis).

Differences between the groups in the utilisation of verbal hostility were evident. Parents with psychosis sometimes responded with name‐calling when hurtful comments were directed at them. Their responses also appeared to be associated with stronger emotional reactions and a struggle to remain calm, which were often influenced by their own mental health and external stressors, highlighting the complexities and challenges they face while navigating their parenting responsibilities.

I shout back sometimes because I get annoyed… I get really upset by all these voices going on and on and on, and like “mummy, this” and it's not her, but it's her that's getting blamed for it, for being in my face and shouting at me, but it's not her that's upsetting me (P46, parent with psychosis).

##### Subtheme 2.3: Non‐reasoning/punitive

In both groups, parents utilised punitive measures, including confining their child to their bedroom and confiscating belongings as disciplinary actions without providing an explanation or a rationale. Some parents mentioned sending their child to their room to prevent losing control or reacting harshly, emphasising the need for personal space and self‐regulation: ‘I have to send her out the room sometimes ‘cause she physically winds me up that much that I feel like I'm going to snap’ (P49, parent with psychosis). For most parents, this approach served mainly to regain composure. For others, isolating their child in their room was a disciplinary measure to separate them from the rest of the household: ‘Sometimes I will actually just go make him sit in his room on his own, because then it's like silent treatment… he can go and sit in his room, because it's not where he wants to be, being on his own’ (P1A, parent without SMI).

There was a clear disparity between the two groups regarding the duration of such punishments. Parents without SMI would typically engage with their child and allow them to return after a brief period, while some parents with psychosis reported not speaking and ‘blanking’ their child for several hours or taking their belongings away for an extended period: ‘he just stayed in his room for a full week… he couldn't have anything no Lego anything so he knows he can't just do that’ (P43, parent with psychosis). Additionally, there were parents in both groups who denied comfort and maintained distance from their child after being told off: ‘Don't even want to look at you or speak to you go away and leave me alone… I thought that's disgusting… and then she's quite upset about it but, this just the way she makes you feel sometimes (P19, parent without SMI).

While both sets of parents employed non‐reasoning and punitive measures, parents with psychosis reported resorting to these methods more frequently.

Although many parents used non‐reasoning/punitive measures to regain composure, for some it reflected their overall parenting style. These parents asserted their authority within the parent–child relationship and established clear boundaries that should be followed. This approach was exemplified by the use of authoritative phrases, such as ‘I am the boss, no, can't have it’ (P46, parent with psychosis) and ‘I have said, you're doing it because I told you to’ (P12, parent without SMI).

#### Theme 3: Permissive/Avoidant Parenting

This parenting style is characterised by a lack of clear boundaries and discipline. Within this theme, made up of two subthemes, parents described either withdrawing from a situation and not responding to their child's behaviour or being indulgent and lenient with their parenting style, giving in to their child's demands and not setting or maintaining rules or expectations. Like authoritarian parenting, this parenting style was reported much less frequently than positive parenting strategies by both groups, and permissive/avoidant parenting was reported much more frequently by parents with psychosis.

##### Subtheme 3.1: Indulgent parenting

Parents in both groups resorted to indulgent parenting, involving ignoring their child's problematic behaviour, not setting rules or boundaries, or ‘giving in’ to their child's desires. Some parents described initially setting rules but later giving in to avoid causing a ‘scene’, considering it not worthwhile. Parents in both groups recounted instances when they completed tasks their child had refused to do. One parent described moving their child's plate to avoid argumentative behaviour: ‘He just shouts no, no, no… I just move it, so I don't have to argue with him’ (P18, parent without SMI). Other parents attributed their permissive behaviour to feeling sorry for their child when they became upset, relating it to their maternal instincts: ‘I let things slide, obviously because it I think it… Mum's do that don't they, I feel sorry for him when he starts crying’ (P32, parent with psychosis).

Some parents with psychosis expressed feeling less in control and would relax their rules more readily, often agreeing with their child to avoid confrontations: ‘I don't feel in control with E… I know the things that like triggers him off and I try and avoid it by agreeing with him and obeying what he wants to do’ (P32, parent with psychosis). Alongside their perceived lack of control, many parents appeared to have resigned themselves to their child's behaviour and relaxed their boundaries due to feelings of inefficacy. They described ignoring their child's misbehaviour because they believed it served no purpose and they had become accustomed to it: ‘I just ignore it… I'm like used to it now and the more I try and resolve it, the worse it gets’ (P54, parent with psychosis). Most parents recognised their leniency in setting boundaries but also acknowledged its long‐term ineffectiveness: ‘I probably don't manage it that well ‘cause it keeps happening… so maybe I don't set firm enough rules… I've always been a bit lax like that’ (P9, parent without SMI).

Parents in both groups were self‐critical and expressed a feeling of being judged by others, which influenced their parenting style and implementation of boundaries. For example, one parent found it difficult to ‘just ignore’ behaviours when other people were present. Although both groups acknowledged the inappropriateness of giving in to their child's behaviour, parents with psychosis appeared to be more critical of themselves, using terms such as ‘I am weak’ (P35), ‘I am too soft’ (P33) and ‘I am really rubbish’ (P54), to describe their parenting skills.

Parents with psychosis reported their mental health having a substantial impact on their ability to enforce boundaries or rules, emphasising the intricate relationship between parental mental health and parenting practices. They found themselves torn between managing their own well‐being and maintaining discipline for their children:

Since I've been a bit not well I've not been shouting a lot because I realise that makes me get into that state that… I've found myself just like accepting it for what it is, I just can't be bothered… and that's where the problem comes into it, I'm just sweeping it under the carpet and its building up and building up (P43, parent with psychosis).

In conclusion, parents in both groups occasionally adopted indulgent parenting but parents with psychosis were much more likely to resort to indulgent parenting practices and described lower self‐efficacy and had heightened self‐judgement regarding their parenting, impacting upon their motivation to instil rules and boundaries.

##### Subtheme 3.2: Withdrawal

Parental accounts showed that both groups of parents used withdrawal as a parenting strategy to help them cope, manage their emotions and prevent behaviours from escalating. This approach was used when parents experienced feelings of anger, frustration or distress, prompting them to take themselves away to a separate room to take ‘deep breaths’ (P16, parent without SMI) or ‘get their head together’ (P31, parent with psychosis). For instance, a parent described the need to step away, saying, ‘I just like go in the other room and breathe, you know like, take a breath and think’ (P37, parent with psychosis). This withdrawal allowed parents to regain composure before re‐entering situations calmly, avoiding shouting or reacting harshly: ‘I tend to just leave her room and take a deep breath, sometimes get a glass of water… just say I don't want to shout at her’ (P16, parent without SMI). Some parents mentioned that they were more likely to withdraw when they were fatigued or stressed, recognising the importance of taking a few moments to prevent overwhelming emotional responses: ‘I have to leave the room and take a few seconds or minutes, because I just think you are doing my head in today, because we're all human and I get to the point sometimes where I'm tired or if I'm due on my period’ (P9A, parent without SMI).

A small number of parents in both groups found withdrawing particularly effective during their child's tantrums or arguments because they felt their involvement would only escalate the situation: ‘If she goes to have a little epi‐fit if you acknowledge that it would last longer than what it would if you just left it, walked away from it’ (P19, parent without SMI). However, there were varying perspectives, with some parents expressing concerns about potentially exacerbating the situation: ‘I probably make things worse because I end up getting really frustrated myself to the point where like I said I just walk out of the room’ (P32, parent with psychosis).

While both groups acknowledged experiencing strong emotions and resorting to leaving the room, a distinct contrast emerged in the intensity of emotional responses, with parents with psychosis expressing more heightened emotional responses. One parent vividly described the necessity of locking themselves in a room, likening the experience to being ‘mashed’ (P32) in the brain and unable to cope. Parents with psychosis highlighted the overwhelming nature of their mental health problems, emphasising how it could ‘get on top of them’, leading to a sense of feeling ‘strangled’ or ‘suffocated’ (P37), and wanting to leave and not return, with one parent stating, ‘I could throw myself off a bridge by the end of the school holidays’ (*P32*). Other parents described the toll of their emotional response to their child's distress: ‘my head starts to pound and my ears start popping… my anxiety levels go up really high and my stress levels and I have to walk out of the room because I end up schitzing out then’ (P42); ‘I think because of my own mental state as well it gets me I mean there's loads of times when he like that I end up in tears and I'll end up frustrated you know where I bite my hand… and that's the time where I think I'm just gonna take all my medication’ (P32).

## DISCUSSION

This study, the first of its kind, explored parenting practices by drawing on a qualitative reanalysis of an existing data set supplemented by additional data, with a focus on exploring potential differences in three key parenting styles. The findings highlighted that most parents aspired to authoritative parenting, emphasising responsiveness, warmth and connection with their children. Parents recognised the importance and effectiveness of these strategies but also reported difficulties in consistently offering an authoritative approach, leading them to occasionally resort to permissive or authoritarian parenting strategies. This study revealed that parents with psychosis are more likely to engage in permissive and authoritarian parenting styles compared to parents without SMI, impacted by factors such as lower self‐efficacy, energy levels, emotional regulation challenges and mental health difficulties. A stark contrast between the groups was evident in parental accounts in terms of parental confidence and ability to cope in the parental role. Consistent with Strand et al. (2020), parents with psychosis identified factors such as stress, energy and fatigue playing an influential role in their ability to offer a consistent parenting style. These factors often hindered their ability to employ reasoning and regulation strategies, maintain composure and refrain from resorting to using authoritarian methods like physical coercion, verbal hostility and non‐reasoning/punitive practices, resulting in feelings of guilt and shame. Unsurprisingly and in line with previous research (Campbell et al., 2012; Harries, Smith, Gregg, & Wittkowski, 2023), parents with psychosis faced additional challenges, including medication side effects and difficulties managing their mental health, leading them to more readily utilise authoritarian and permissive strategies. However, despite these challenges, both sets of parents prioritised spending time with and expressing affection towards their children, highlighting the resilience and dedication of parents across both groups.

Similarly, although both sets of parents were just as likely to establish rules and boundaries, parents with psychosis encountered greater difficulties in enforcing and consistently following through with these rules, as well as implementing structure and routines. Lower self‐efficacy and heightened self‐criticism further hindered their ability to maintain boundaries, often resulting in the implementation of a more permissive parenting style, consistent with findings from previous reviews of parents with SMI (Harries, Smith, Gregg, & Wittkowski, 2023) and psychosis (Collins et al., 2025). Within the current study, the profound emotional burden encountered by parents with psychosis was evident because they discussed the necessity of withdrawing from parenting situations. Withdrawal served as a coping mechanism and to prevent behaviours from escalating for both sets of parents, which has been found to be a common response to stress in difficult parenting situations (Szymańska & Dobrenko, 2017).

However, parents with psychosis displayed heightened emotional responses, struggled with coping, and frequently felt the need to disengage from situations. These findings align with the ABC‐X model of family stress (Rosino, 2016), which highlights how stressors, a lack of coping resources, and support can lead to maladaptive family functioning. As parents with psychosis are more vulnerable to stress (Campbell et al., 2018; Radley, Barlow, & Johns, 2022; Zubin & Spring, 1977) and are less likely to have access to support (David et al., 2011; Day et al., 2011; Engur, 2017), it is unsurprising that they are more likely to withdraw to cope. Additionally, these findings enhance previous research that highlights how psychosis can impact a parent's ability to cope with the parental role (Campbell et al., 2018; Harries, Smith, Gregg, Allott, & Wittkowski, 2023; Harries, Smith, Gregg, & Wittkowski, 2023; Strand et al., 2020).

### Clinical implications and recommendations

In line with recommendations urging increased efforts to support families affected by parental mental illness (Diggins, 2011; Radley, Sivarajah, et al., 2022), this study emphasises the need for promoting conversations regarding parenting within adult mental health services and recognising the significance and difficulty of the parental role for parents with psychosis. Support for these parents should also recognise that they are more likely to be parenting solo and potentially lacking familial support. The stress of parenting is also likely to be having a detrimental impact on their mental health. Our findings illustrate that parents with psychosis may benefit from evidence‐based parenting programs. Parents reported experiencing guilt when resorting to an authoritarian parenting style, suggesting that emotional regulation strategies may help them feel more in control and composed when parenting, potentially leading to a more consistent and authoritative approach. By focusing on building parenting skills and self‐efficacy, there is potential to increase parental confidence in applying rules and boundaries, thereby reducing the tendency to employ permissive parenting strategies due to feeling inadequate or overwhelmed. One such program is the ‘Triple P’ positive parenting program (Sanders, 1999), which includes strategies for managing emotional responses, building parental confidence, enhancing parental self‐care (managing stress) and promoting consistent and positive parenting approaches (Sanders et al., 2000). There is preliminary evidence (Wolfenden et al., 2022) that Triple P can improve parental mental health (including positive psychotic symptoms) and parenting efficacy in parents with psychosis. Considering the significant impact of parenting challenges for the whole family (Abel et al., 2019; Davidsen et al., 2022; Pierce et al., 2020), interventions should encompass a whole family approach to mitigate the risks associated with parental mental health difficulties (Diggins, 2011; Foster et al., 2016; Harries, Smith, Gregg, & Wittkowski, 2023; Moltrecht et al., 2024), which is currently scarce within services (Tuck et al., 2023).

### Strengths and limitations

The sample size of 40 parents and inclusion of parents without SMI are strengths of the study and have allowed for in‐depth exploration of the similarities and differences between the two groups of parents. The detailed and in‐depth nature of the CFI allowed for a candid exploration of key areas of family life, providing greater insight into parents' day‐to‐day practices. Although a wide range of parenting practices was discussed, more specific questions on parental consistency, neglectful parenting or the use of observations may be helpful for future research. A fourth parenting style ‘neglectful parenting’ later added by Baumrind (1989), was incorporated in the initial template but later excluded due to its infrequent occurrence in the transcripts. While using the CFI allowed for candid exploration and reduced the potential pressure of parents feeling judged or the need to express socially desirable parenting practices, it is important to consider that parenting can be a sensitive topic, and parents express feeling judged (Simmons, 2020). Parents expressed that they often feel guilty for using permissive or authoritarian parenting styles, which may have caused parents to emphasise more socially desirable responses, impacting the findings.

Evidence suggests that parents with psychosis are more likely to experience greater adversity and to be unemployed (Boydell et al., 2013; Craig & Bromet, 2004; Topor et al., 2016), and although attempts were made to match the groups on key demographic characteristics, the sample of parents with psychosis was less likely to be employed, and as a result, experienced greater financial difficulty. There also appeared to be a higher incidence of developmental delay in the children of parents with psychosis (albeit non‐significant statistically). These differences may have impacted parental stress levels, ability to cope and potentially the findings.

Both groups were matched on gender and ethnicity; most parents were mothers (95%) and white British (80%), leading to a possible gender bias and cultural influence, limiting the transferability of the findings to other parenting populations and fathers. Future research using a sample with more diverse backgrounds, closely matched on socio‐political contexts, would be beneficial in order to enable wider conclusions to be drawn.

## CONCLUSIONS

Our findings make a significant contribution to the limited qualitative research exploring parenting practices among parents with psychosis. The findings of this study highlight the frequent use of positive authoritative strategies by both groups of parents, alongside the additional challenges parents with psychosis face, impacting their ability to consistently employ authoritative practices. Parents who experience psychosis would benefit from additional support through family‐focused approaches and evidence‐based parenting courses, focusing on emotional regulation techniques, teaching parenting skills and improving self‐efficacy.

## AUTHOR CONTRIBUTIONS

**Hannah Collins:** Investigation; writing – original draft; formal analysis; data curation. **Anja Wittkowski:** Writing – review and editing; supervision. **Lauren Wolfenden:** Investigation; data curation. **Rachel Calam:** Supervision. **Lynsey Gregg:** Conceptualization; writing – review and editing; methodology; supervision; formal analysis.

## CONFLICT OF INTEREST STATEMENT

The authors declare no conflicts of interest.

## Data Availability

The data that support the findings of this study are available upon request from the corresponding author. The data are not publicly available due to privacy or ethical restrictions.
